# Exploratory study to assess feasibility of intracerebral hemorrhage detection by point of care cranial ultrasound

**DOI:** 10.1186/s13089-022-00289-z

**Published:** 2022-10-17

**Authors:** Aarti Sarwal, Yash Patel, Ralph D’Agostino, Patrick Brown, Stacey Q. Wolfe, Cheryl Bushnell, Casey Glass, Pamela Duncan

**Affiliations:** 1 Neurology, Wake Forest School of Medicine, Winston-Salem, USA; 2 Wake Forest School of Medicine, Winston-Salem, USA; 3 Biostatistics and Data Science, Clinical Research Management Graduate Program, Biostatistics Shared Resource, Comprehensive Cancer Center, Wake Forest School of Medicine, Winston-Salem, USA; 4 Neurology and Radiology, Wake Forest School of Medicine, Winston-Salem, USA; 5 Neurosurgery, Wake Forest School of Medicine, Winston-Salem, USA; 6 Emergency Medicine, Wake Forest School of Medicine, Ultrasound Integrated Curriculum, Winston-Salem, USA

**Keywords:** Brain hemorrhage, Echography, Ultrasound imaging, Sonography, Doppler transcranial

## Abstract

**Background:**

Limited studies have evaluated the use of ultrasound for detection of intracerebral hemorrhage (ICH) using diagnostic ultrasound Transcranial Doppler machines in adults. The feasibility of ICH detection using Point of care Ultrasound (POCUS) machines has not been explored. We evaluated the feasibility of using cranial POCUS B mode imaging performed using intensive care unit (ICU) POCUS device for ICH detection with a secondary goal of mapping optimal imaging technique and brain topography likely to affect sensitivity and specificity of ICH detection with POCUS.

**Materials and methods:**

After obtaining IRB approval, a blinded investigator performed cranial ultrasound **(**Fujifilm, Sonosite^®^ Xporte, transcranial and abdominal presets) through temporal windows on 11 patients with intracerebral pathology within 72 h of last CT/MRI (computed tomography scan/magnetic resonance imaging) brain after being admitted to a neurocritical care unit in Aug 2020 and Nov 2020–Mar 2021. Images were then compared to patient’s CT/MRI to inform topography. Inferential statistics were reported.

**Results:**

Mean age was 57 (28–77 years) and 6/11 were female. Six patients were diagnosed with ICH, 3 with ischemic stroke, 1 subarachnoid hemorrhage, and 1 brain tumor. The sensitivity and specificity of point of care diagnosis of ICH compared to CT/MRI brain was 100% and 50%, respectively. Mean time between ultrasound scan and CT/MRI was 13.3 h (21 min–39 h). Falx cerebri, choroid calcification and midbrain-related artifacts were the most reproducible hyperechoic signals. Abdominal preset on high gain yielded less artifact than Transcranial Doppler preset for cranial B mode imaging. False positive ICH diagnosis was attributed to intracerebral tumor and midbrain-related artifact.

**Conclusions:**

Our exploratory analysis yielded preliminary data on use of point of care cranial ultrasound for ICH diagnosis to inform imaging techniques, cranial topography on B mode and sample size estimation for future studies to evaluate sensitivity and specificity of cranial POCUS in adult patients. This pilot study is limited by small sample size and over representation of ICH in the study. Cranial POCUS is feasible using POCUS machines and may have potential as a screening tool if validated in adequately powered studies.

**Supplementary Information:**

The online version contains supplementary material available at 10.1186/s13089-022-00289-z.

## Background

Limited studies have evaluated the use of ultrasound for detection of intracerebral hemorrhage (ICH) using diagnostic high end transcranial color-coded duplex machines. [1–5] B mode imaging, in particular, has been evaluated for tumors, movement disorders, midline shift and hydrocephalus assessment, but there is no published literature on imaging techniques and parenchymal topography for cranial B mode imaging [6–10]. Technological advances in point of care ultrasound (POCUS) devices have allowed increasing use of ultrasound as a diagnostic modality in critical care and emergency settings as investigations reveal similar diagnostic accuracy for diseases such as ascites, pleural effusion, and aortic aneurysm but studies assessing feasibility and accuracy of cranial POCUS to diagnose intracerebral pathology such as ICH are sparse. [11, 12] A recent study in pediatrics explored use of cranial POCUS in pediatric head trauma but was confounded by pediatric skull characteristics that are different than adults due to temporal bone thickening [13]. With increasing use of POCUS in neuro-emergencies, it is a natural progression to investigate the use of POCUS in diagnosis of intracerebral pathology. The increasing recognition can be fathomed by inclusion of ICH detection by brain ultrasound as a pre-advance or advance skill by consensus of neuroultrasound experts in intensive care [14]. Investigations into accuracy of cranial ultrasound imaging and topography informing ICH detection can provide avenues for early field diagnosis of ICH.

We evaluated the feasibility of cranial POCUS B mode imaging performed using an intensive care unit (ICU) POCUS device for ICH detection with a secondary goal of informing optimal imaging techniques and mapping brain topography of B mode images. We used this study to characterize anatomy and pathology that would affect the sensitivity and specificity of ICH detection with POCUS to generate preliminary data for future studies assessing diagnostic accuracy of POCUS in ICH detection in comparison with CT brain scan.

## Methods

### Subjects

After obtaining IRB approval, we prospectively screened adult patients (≥ 18 years) with any intracerebral pathology admitted to the neurocritical care unit at a tertiary care academic medical center in Aug 2020 and then Nov 2020–March 2021 who were anticipated to stay in the intensive care unit for > 72 h. The study recruitment period reflects the impact of pandemic-imposed research enrollment restrictions. Consent was obtained in person from the legally authored representative if patients were unable to consent. Electronic consents were not available for research trials during this study recruitment period and visitor restrictions were in place due to COVID. Patients were included if they had cerebral imaging with CT or Magnetic Resonance Imaging (MRI) performed as part of standard of care and an investigatory ultrasound could be performed within 72 h of the CT or MRI scan. Patients were excluded if they had any penetrating head trauma or skull defects that could affect ultrasound insonation, if they had isolation precautions in effect for suspected or confirmed COVID infection and if images could not be obtained within 72 h of last CT or MRI imaging.

### Design

All scans were performed by an investigator (AS) with experience in neuroimaging, diagnostic neurovascular sonography and point of care ultrasound. The investigator performed B mode imaging only when able to be completely blinded to the patient’s diagnosis and not participating in direct patient care. A report was logged by the investigator immediately after the ultrasound scan to report ultrasound findings, namely, if the patient had an intracerebral lesion suspicious for intracerebral hemorrhage based on the presence of hyperechoic signal at a location not corresponding to an expected intracerebral anatomical structure. Stored images were reviewed post-hoc by the same investigator with comparison to patient’s CT scans for elucidating B-mode pathology corresponding to signals seen on ultrasound. Knowledge gained from each review informed future imaging. For example, choroid plexus was deemed to cause false positive findings by mimicking possible hemorrhage. After comparison with CT scan, the location of hyperechoic signals in relation to each other, such as the midbrain, provided the expected visualization for future scans to note presence of choroid plexus. All images were serially reviewed with a faculty neuroradiologist (PB) to define different anatomical structures visualized on cranial ultrasound and explore the correlative anatomy and artifacts seen on a cranial ultrasound. We considered this step necessary, since there was no available resource on cranial topography as a reference beyond the basic anatomy of visualizing the midbrain, sphenoid wing and petrous part of the temporal bone commonly used for transcranial color-coded duplex imaging. Since we reviewed ultrasound images and corresponding CT scans after every image acquired in an iterative fashion, each scan served as a learning tool for follow-up scans helping readjust interpretation.

### Ultrasound imaging

A low frequency 3–1 MHz phased array probe (echo probe) on ICU POCUS device (Fujifilm, Sonosite® Xporte) was used to perform B mode images of the brain using the temporal windows by the investigator blinded to patient’s diagnosis. Transcranial and abdominal presets were used to visualize the opposite skull visible as a hyperechoic shadow. A depth of 13–16 cm was used and adjusted to ensure the opposite skull could be visualized. Probe index marker was pointed toward the eyes with line of insonation aligned to get an axial section of the brain. Transcranial preset was used due to high mechanical and thermal index designed to facilitate imaging through the skull. Abdominal presets were investigated to allow future scalability of this feasibility study on hand-held machines, most of which don’t have transcranial presets. The midbrain was visualized as a butterfly shaped structure. The exam was repeated on each side to assess for presence of a temporal window. The patient was excluded if neither temporal windows were present. Once adequate windows were confirmed with both the skull and midbrain visualized, the probe was pointed above and below the level of midbrain and then positioned to look for any reproducible signals corresponding to expected anatomical structures or abnormal pathology. Signals corresponding to a lesion or anatomical structure were labelled as “hyperechoic” if the lesion/structure appeared white (or bright) with echogenicity similar to the echoic character of the skull visible on B mode.

### Analysis

We present a descriptive analysis of patient demographics and ultrasound imaging characteristics visible on cranial ultrasound in relation to its ability to detect ICH. We evaluated images for presence of anatomical landmarks, brain topography and alternative etiologies that could potentially affect sensitivity and specificity of an ICH diagnosis using point-of-care cranial ultrasound. We compared the results of using static images on B mode ultrasound as well-recorded cine loops. Acquisition of images using the abdominal preset was compared to findings on the transcranial Doppler preset. Inferential statistics were used to generate exploratory data on sensitivity, specificity and accuracy of POCUS diagnosis of ICH in comparison with CT scan diagnosis of ICH. We calculated 95% exact confidence intervals using Jeffrey’s intervals due to the small sample size [15]. The exploratory nature of this study design and small sample size prohibited meaningful analysis of impact of age, time since onset, or comparison of CT Hounsfield units to ultrasound detection of hemorrhage. The STROBE checklist was used to report findings, since feasibility was the initial aim with secondary aim to describe imaging technique and topography relevant to ultrasound-based ICH detection.

### Results

A total of 30 patients were eligible during the screening period. Consent could not be obtained in 10 patients despite documented temporal windows due to inability to locate or contact legally authorized representative to obtain consent. Three patients had no temporal windows. Of the 17 consented with temporal windows, 4 patients could not receive an ultrasound during their hospital stay due to death, discharge or sonographer unavailability, and 2 patients had expected concurrent CT/MRI imaging delayed, thus the ultrasound scans occurred ≥ 72 h from last CT/MRI. Ultrasound images were obtained and saved successfully for review in 11 patients (Table 1). These patients had a mean age of 57.45 years (11 patients, 28–77 years, 5 males). The mean time between CT/MRI and ultrasound was 13.3 h (21 min–39 h). 7 patients had some form of intracranial hemorrhage reported on CT brain (one with hemorrhagic conversion within ischemic stroke one with SAH but interhemispheric bleed), 3 patients had ischemic stroke without (Table 2) any hemorrhagic conversion, and 1 patient had a thalamic tumor.

**Table 1:**
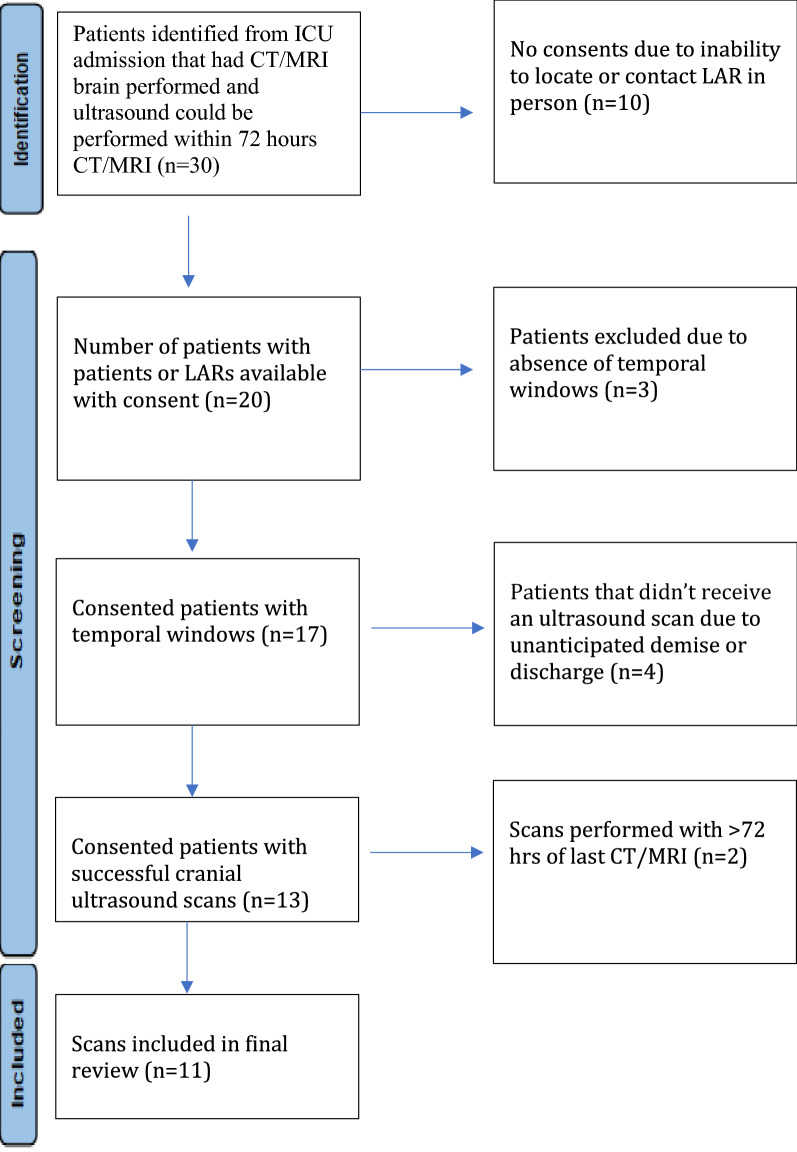
Flow diagram of study enrolment

From: Page MJ, McKenzie JE, Bossuyt PM, Boutron I, Hoffmann TC, Mulrow CD, et al. The PRISMA 2020 statement: an updated

**Table 2:** Patient and imaging characteristics from CT scan and post hoc ultrasound analysis of enrolled patients who had temporal windows

Subject	Age, gender		Diagnosis based on CT/MRI brain	Ultrasound lesions on Transcranial present in comparison with head CT	Ultrasound lesions on Abdominal present in comparison with head CT	Time b/w CT/MRI & ultrasound scan	POCUS blinded to CT diagnosis outcomes ICH
1	28 male	AIS	Large subacute left MCA distribution infarct No hemorrhagic conversion external ventricular drain right frontal lobe and tip terminates in the right frontal horn	Posterior part of falx cerebri (Figs. 2, 3, 6) Choroid plexus midline and temporal horns (Fig. 2) Linear hyperechoic shadow parallel to inner table posterior fossa) ~ transverse sinus)	Posterior part of falx cerebri Choroid plexus midline and temporal horns Linear hyperechoic shadow parallel to inner table posterior fossa) ~ transverse sinus)	1 day, 7 h	True negative
2	55 female	Tumor	Peripherally enhancing lesion in the left thalamus 3.5 × 3.3 cm	Large Hyperechoic shadow below midbrain (Fig. 5)	Discrete delineated Hyperechoic shadow below midbrain	1d 15 h	False positive
3	35 female	AIS	Large right early subacute MCA territory infarcts. No hemorrhagic transformation Early subacute right thalamic infarct Punctate hyperdense focus in the high peripheral right frontal lobe may represent a prominent surface vessel versus punctate hemorrhage	Large Hyperechoic shadow below midbrain (Fig. 4)	Echogenicity in anterior and posterior temporal lobe Echoic cisterns around midbrain	3 h	False positive
4	54 female	ICH	Right basal ganglia hemorrhage with intraventricular extension Obstructive hydrocephalus of the left ventricle. Extension of the hemorrhage into the anterior horn, occipital horn, and body of the right lateral ventricle, third ventricle, and fourth ventricle	Posterior part of falx cerebri (Figs. 2, 3, 6) Large hyperechoic shadow cortical Sphenoid bone wings (Fig. 1)	Posterior part of falx cerebri Hyperechoic shadow subcortical Sphenoid bone wings	13 h	True positive
5	63 male	ICH	Right basal ganglia intraparenchymal hemorrhage No intraventricular hemorrhage	Hyperechoic shadow subcortical ( Fig. 3) Hyperechoic shadow below midbrain Linear hyperechoic shadow parallel to inner table posterior fossa) ~ transverse sinus)	hyperechoic shadow subcortical Hyperechoic shadow below midbrain Linear hyperechoic shadow parallel to inner table posterior fossa) ~ transverse sinus)	14 h	True positive
6	69 male	ICH	Right thalamic hemorrhage w Hemorrhage layering dependently within the occipital horns of the lateral ventricles. Severe atherosclerotic calcifications of the intracranial internal carotid arteries Chronic microvascular ischemic changes	Very hyperechoic shadow subcortical (Fig. 3)	Very hyperechoic shadow subcortical	21 min	True positive
7	64 female	SAH	Endovascular coiling of ruptured anterior communicating artery aneurysm. Extra-axial hemorrhage along the interhemispheric fissure and decreasing subarachnoid hemorrhage throughout the bilateral sylvian fissures and frontal lobe sulci. Extensive patchy hypodensities throughout the bilateral cerebral white matter	Very hyperechoic shadow subcortical	Very hyperechoic shadow subcortical	1 day, 3 h	True positive
8	57 female	ICH	MRI—Acute left parietal parenchymal hematoma measuring 3.1 × 3.0 × 3.4 cm with thin peripheral enhancement, presumably associated with an underlying metastatic lesion	Very hyperechoic shadow subcortical (Fig. 3) Clivus	Very hyperechoic shadow subcortical Clivus Choroid plexus midline and temporal horns	4 h 50 min	True positive
9	77 female	AIS	No acute intracranial hemorrhage or evidence of acute large vascular territory infarct. Chronic small vessel disease. Intracranial atherosclerosis	Choroid plexus midline and temporal horns (Fig. 2)	Choroid plexus midline and temporal horns	7 h	True negative
10	76 male	ICH	Large right MCA territory infarct with leftward midline shift 13 mm. Hemorrhagic conversion of the infarct predominantly involving the right basal ganglia	Very hyperechoic shadow subcortical (Fig. 3) Hyperechoic shadow below midbrain (Fig. 4) Echoic cisterns around midbrain	Very hyperechoic shadow subcortical	6 h 20 min	True positive
11	54 male	ICH	Acute right basal ganglia hemorrhage with mild surrounding edema and local mass effect. Patchy hypodensity within the periventricular and deep white matter	Falx cerebri (Fig. 2,3 &6) Very hyperechoic shadow subcortical (Fig. 3) Choroid plexus midline and temporal horns (Fig. 2)	Choroid plexus midline and temporal horns	47 min	True positive

The blinded ultrasound diagnosis compared to the CT head is provided in the last column

#### Imaging technique

**Fig. 1:**
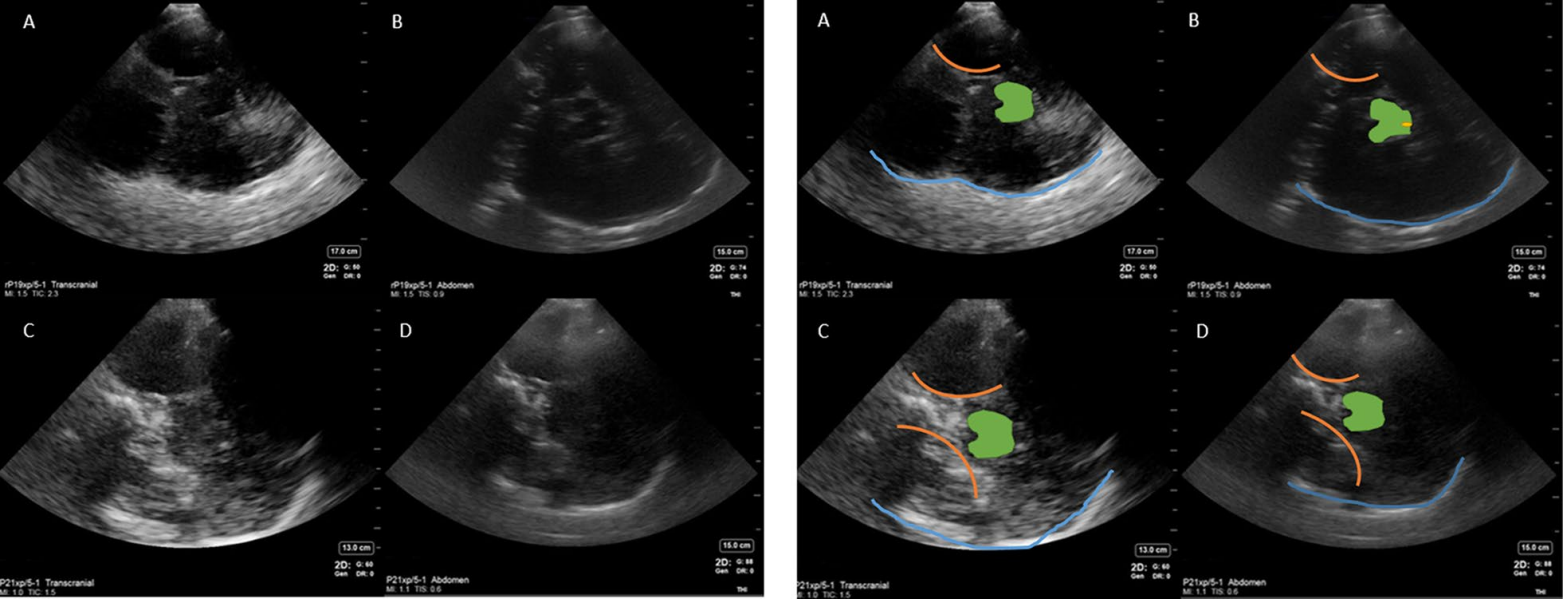
Comparison of cranial B mode images on abdominal and transcranial preset with anatomical landmarks visible. *Right Image Panel* **A**, **C** are transcranial presets and *Right Image Panel* **B**, **D** are abdominal presets on same image. Marking on these images are labelled as followed: *Blue line*—opposite skull, *orange line* sphenoid wing and petrous part temporal bone, *green* midbrain, *orange dot* inside the midbrain—cerebral aqueduct. *Left image* shows unlabeled images of of both the transcranial presets (**A**, **C**) and abdominal presets (**B**, **D**)

We used the low frequency echo probe placed on the patient's temple with index marker of the probe pointed toward the patient’s eyes (Additional file 1: Figure S1). The probe was positioned with the line of insonation aligned with an imaginary line from lateral canthus of the eye to the tragus. Depth of the image was adjusted to allow insonation of the opposite skull typically requiring 13–16 cm depth. Once the skull was visualized, the probe was slowly rocked cranially caudally until the butterfly shaped midbrain was visualized which allowed distinction of ipsilateral and contralateral cerebral hemisphere. We then rocked the probe further cranially and caudally followed by anteriorly and posteriorly to look for other hyperechoic signals. In general, visualization of brain parenchyma was more feasible contralateral to the window insonated (Fig. 1). Hence, left temporal windows were best suited to assess for right-sided parenchymal pathology and vice versa. The sector nature of the probe and artifact produced by ipsilateral sphenoid wing made it difficult to visualize the anatomy ipsilateral to the insonated window. Abdominal presets with high gain settings provided comparable resolution for imaging brain to elucidate known anatomical and abnormal pathological structures when compared to transcranial preset. Transcranial preset tended to produce some artifacts that were not visible in abdominal presets. We did not find any challenges with imaging associated with placement of electroencephalography electrodes or invasive monitoring devices. Rocking the probe from one location did not enable visualization of the whole anterior–posterior span of the skull and we found it useful to move the probe across the temporal bone for maximum visualization, which sometimes led to the loss of temporal window. Realigning the probe to visualize the opposite skull was then re-attempted and the process repeated. The duration of each US was 5–10 min to image both the right- and left-side windows. Sonographer position at the side of the bed was used for all scanning and it was feasible to scan both sides while standing in one location. No patient positioning was necessary for the study scans. Though none of the study patients had significant head movement or agitation during scanning, we did need to adjust the probe for head turning or cough.

#### Topography on cranial ultrasound imaging

**Fig. 2:**
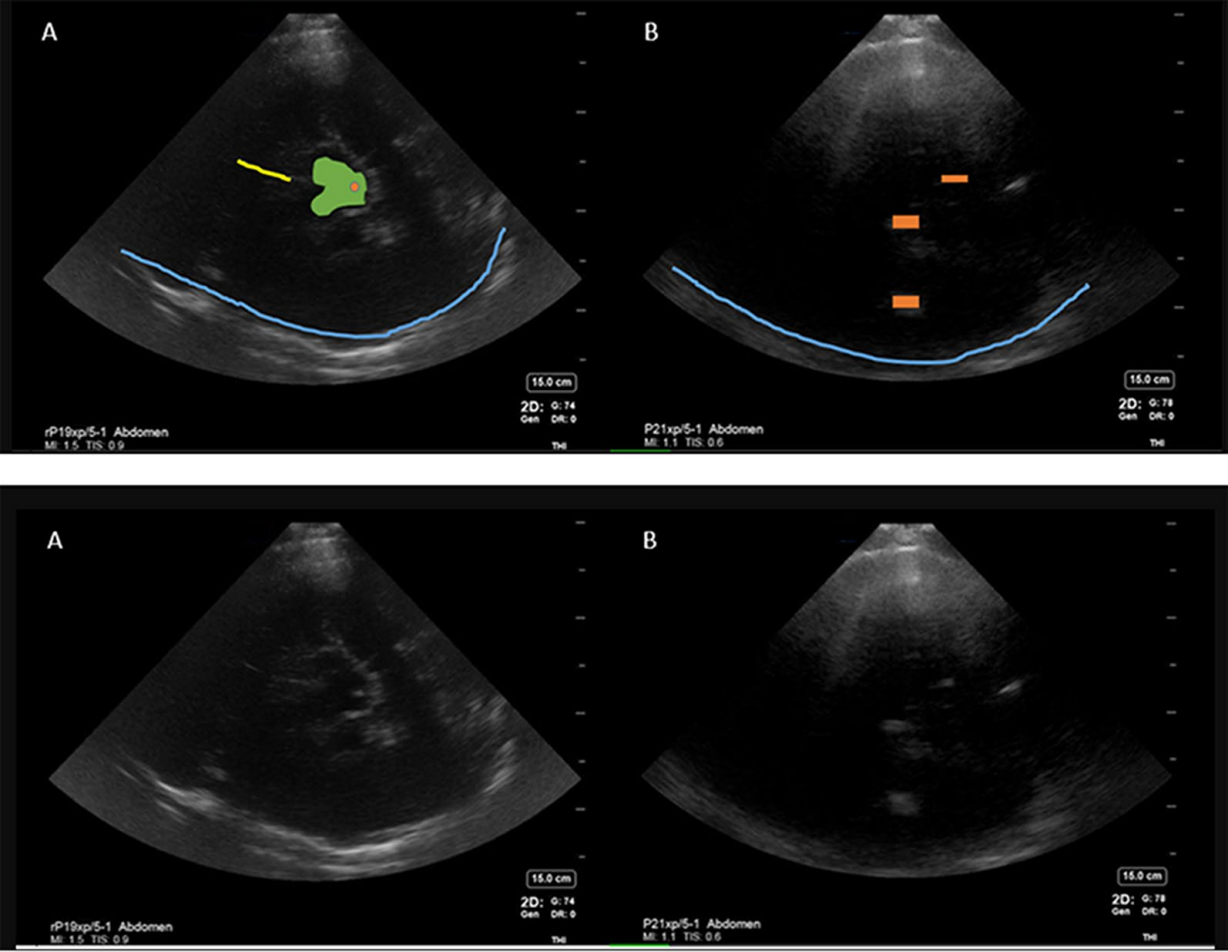
Abdominal presets showing the cerebral aqueduct and choroid plexus on cranial B mode imaging. *Upper panel* shows abdominal presets with markings labelled as followed: *Blue line*—opposite skull, *green*—midbrain, *orange dot* inside the midbrain—cerebral aqueduct, *yellow line*—falx cerebri, orange rectangles—choroid plexus calcification in lateral ventricles. *Lower panel* shows unlabeled images of abdominal presets

The most discernible anatomical structures visible on B mode imaging in patients with windows have been outlined in Figs. 1 and 2 and included:

- Opposite skull visible as a convex hyperechoic signal farthest from the probe. Depth was adjusted to visualized this at the bottom of the image
- Midbrain, visible as a hypoechoic butterfly-shaped structure in the center of the ultrasound image. Peduncles and colliculi were generally visible to allow identification of midbrain.
- Cerebral aqueduct visible as a hyperechoic shadow in the posterior part of the midbrain.
- Sphenoid wings and petrous part of the temporal bone visible as hyperechoic signal, both ipsilateral and contralateral.
- Falx cerebri visible as a linear hyperechoic signal originating from frontal end of the ultrasound images.
- Calcified choroid plexus visible in midline and in the temporal horns of lateral ventricles.

**Fig. 3:**
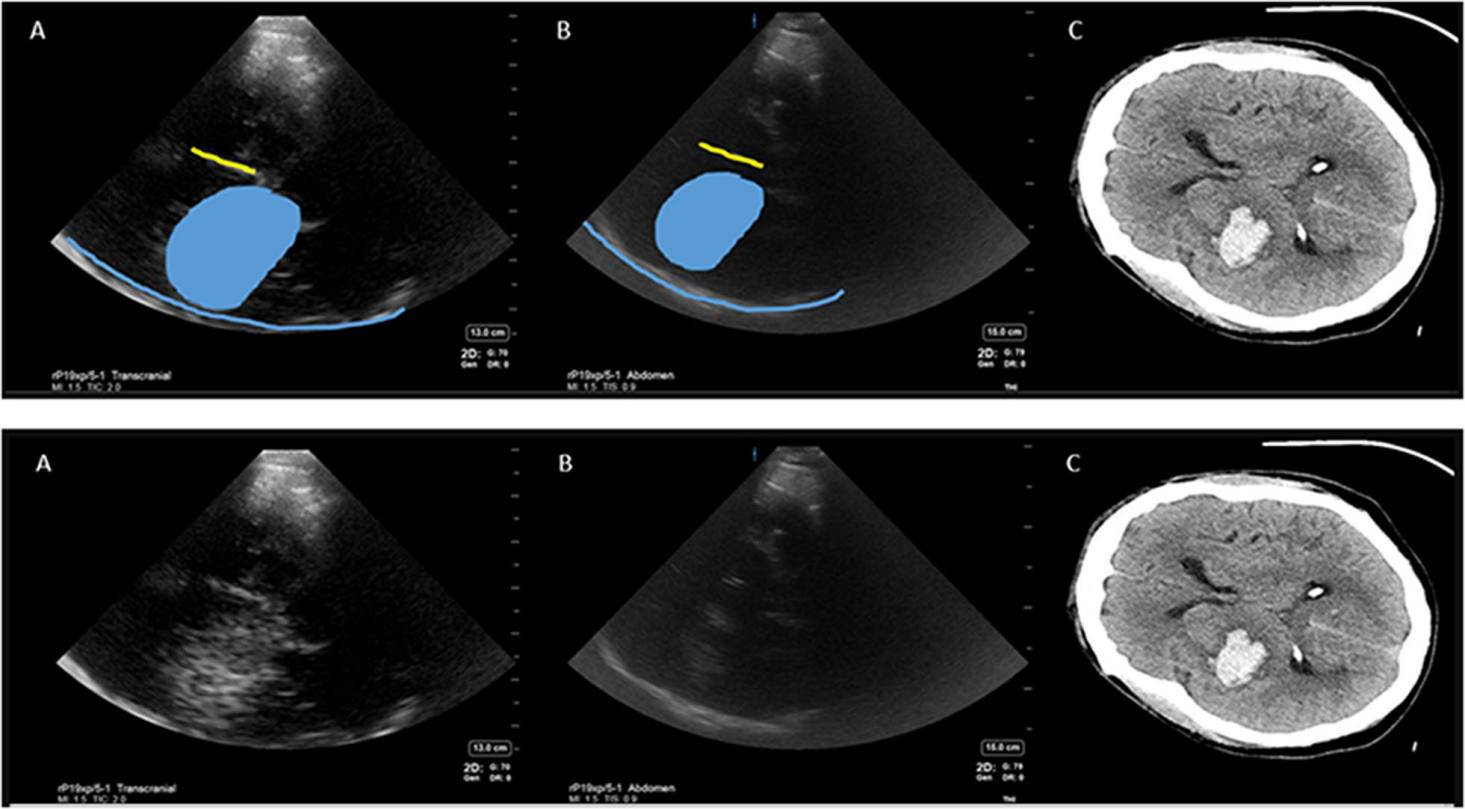
Intracerebral hemorrhage visible as a hyperechoic signal best visualized contralateral to the insonated window. **A**—Transcranial preset, **B**—abdominal preset, **C**—COMPUTED tomography brain (CT) scan. Blue line—opposite skull, yellow line—falx cerebri, blue shape outlines the hyperechoic signal corresponding to hemorrhage on CT scan

**Fig. 4:**
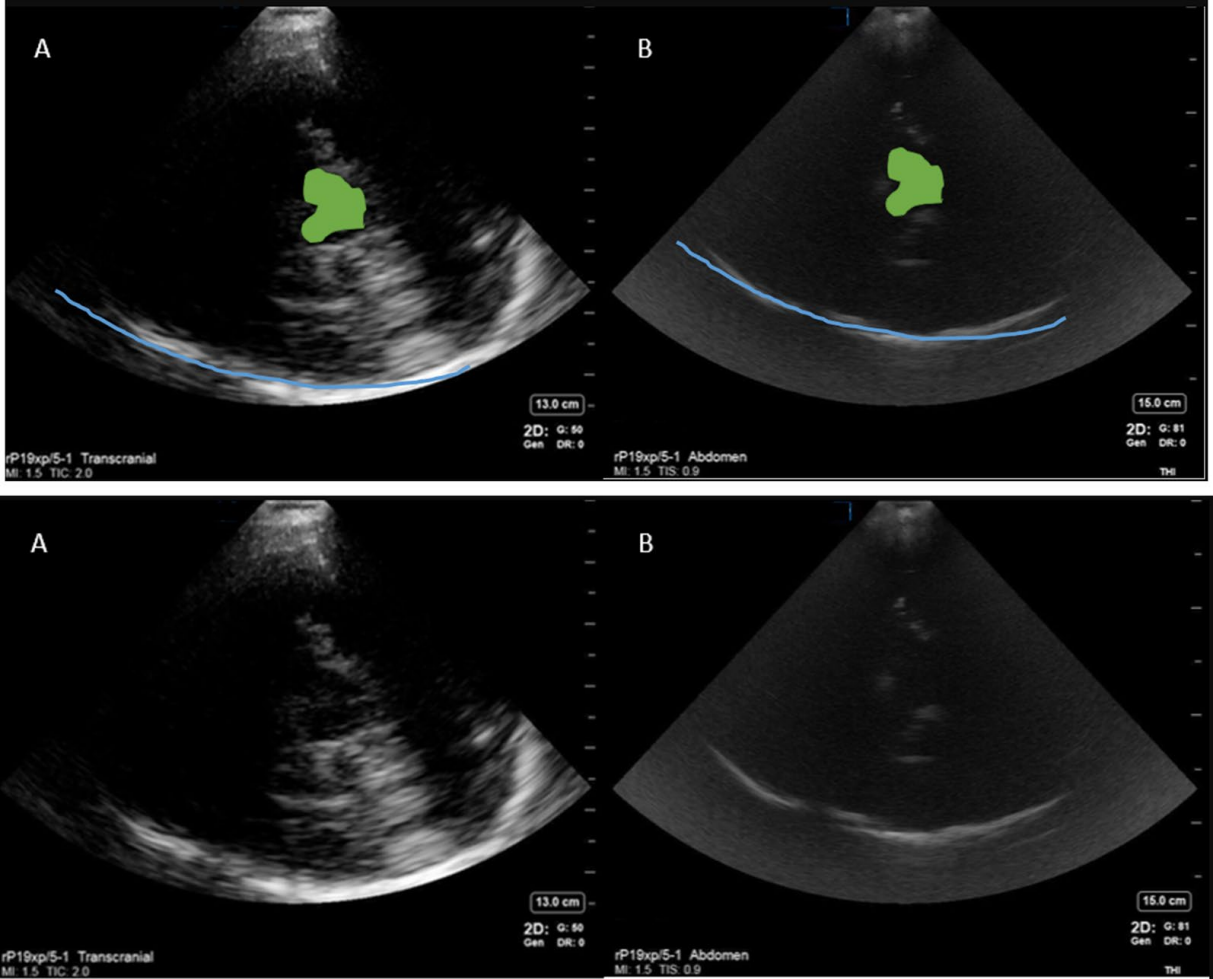
Artifact created by acoustic shadow of the midbrain causing a false positive finding of hemorrhage More visible on transcranial preset **A** but less enhanced on abdominal preset **B**. Blue line—opposite skull, green—midbrain

Suspected hemorrhage could be visualized as a hyperechoic signal reproducible in multiple planes by rocking the probe in anteroposterior and cranial caudal directions at a location, where no other hyperechoicity was expected (Fig. 3). In addition to above anatomical landmarks, the following reproducible hyperechoic artifacts were identified as possible hemorrhage mimics: (Fig. 4).

- Hyperechoic signals in cisterns around the midbrain with signal intensity lower than bone (opposite skull).
- Hyperechoic shadows inferior to the midbrain (midbrain acoustic shadow) could appear similar to hyperechoic signals produced by a hemorrhage. This contributed to one of the earlier false positive findings.
- Hyperechoic signals parallel to the occipital bone causes by thick skull ridges in the posterior fossa.

#### Blinded imaging results

**Fig. 5:**
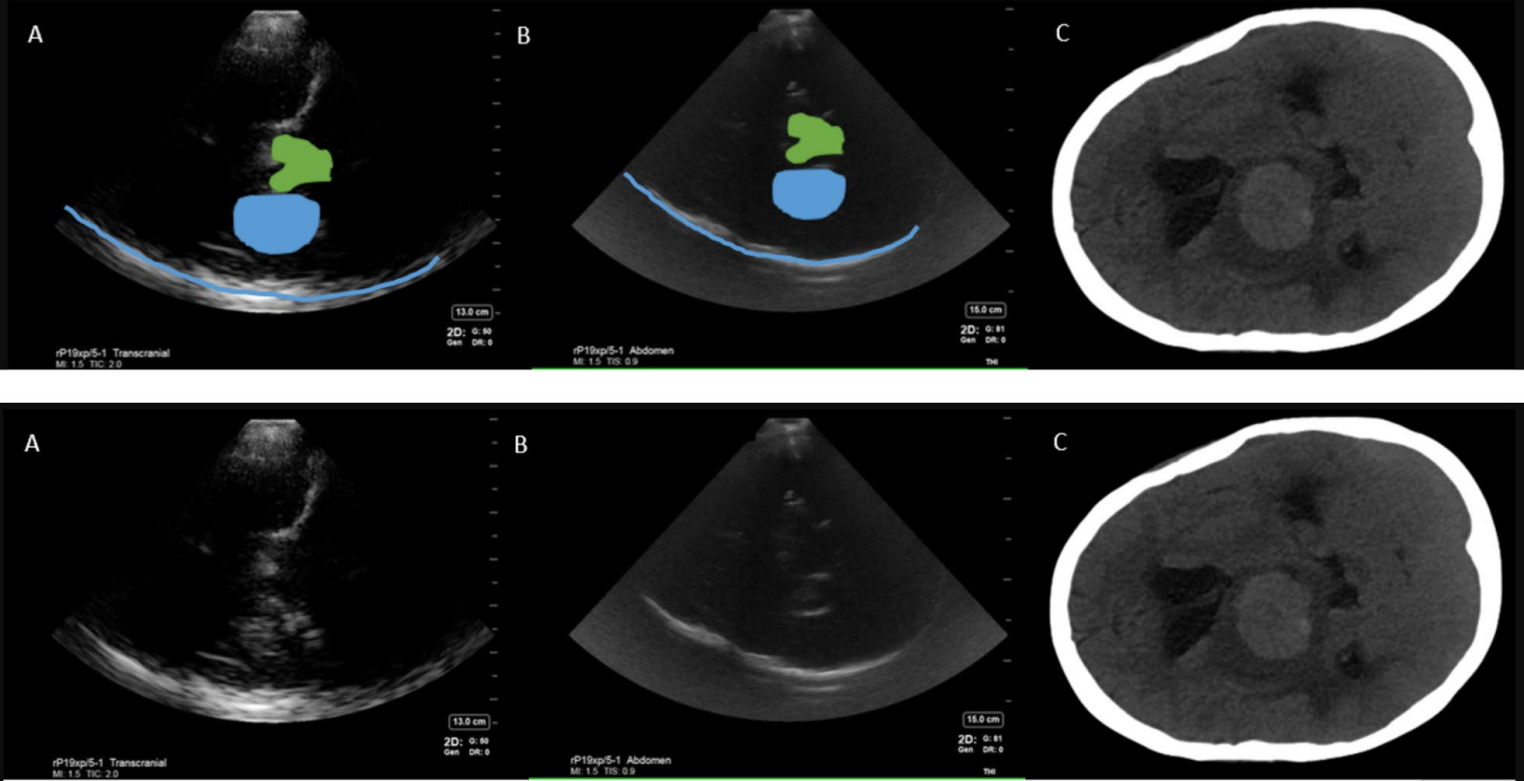
Thalamic tumor creating a hyperechoic signal similar to intracerebral haemorrhage. **A**—Transcranial preset, **B**—abdominal preset, **C**—computed tomography (CT) brain scan. Blue line—opposite skull, green—midbrain. Blue shape outlines the hyperechoic signal corresponding to the tumor on CT scan

**Fig. 6:**
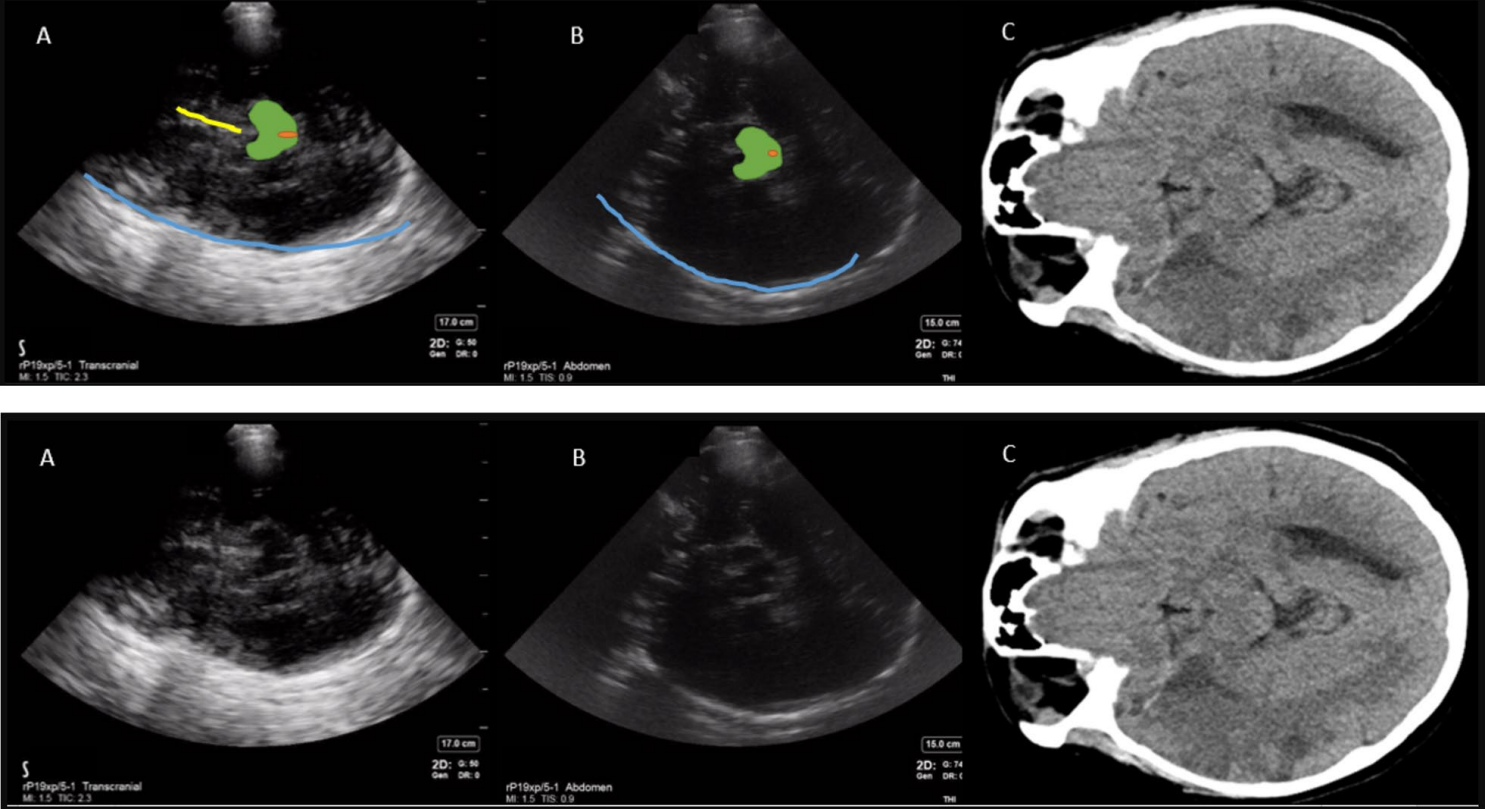
Acute ischemic stroke does not produce a characteristic appearance of ultrasound that allows ultrasound-based diagnosis. **A**—Transcranial preset, **B**—abdominal preset, **C**—computed tomography brain scan. Blue line—opposite skull, green midbrain, orange dot inside the midbrain—cerebral aqueduct, yellow line—falx cerebri

Temporal windows were found in all but 3 patients (18.75%, including two that were excluded from post hoc analysis) which is comparable to published literature [16, 17]. The blinded investigator made a point of care diagnosis of ICH with 100% sensitivity and 50% specificity in comparison with a head CT (Table 3). The hyperechoic signals created by the artifact produced by midbrain acoustic shadows (Fig. 4) and a thalamic tumor (Fig. 5) contributed to the two false positive findings. Ischemic stroke did not create any discernible signals on ultrasound (Fig. 6).

**Table 3:** Results of blinded investigator-based diagnosis using POCUS compared to patient diagnosis

		Diagnosis by neuroimaging (head CT or MRI)		
		Condition positive (ICH present) 6 ICH 1 SAH with ICH	Condition negative (No ICH present) 4	
POCUS result outcome	Positive (ICH present)	True Positive 7	False Positive 2	Positive predictive value 77% = TP/(TP + FP)
	Negative (ICH not present)	False Negative 0	True Negative 2	Negative Predictive value 100% = TN(FN + TN)
		Sensitivity 100% = TP/ (TP + FN)	Specificity 50% = TN/(FP + TN)

#### Post hoc analysis

During post hoc image analysis, static snapshots of B mode images were compared to recorded cine loops by the principal investigator and neuroradiologist. We observed that review of cine loops revealed better delineation of relevant anatomical landmarks as well-reproducible hyperechoic lesions with appearance of a hemorrhage. None of the hyperechoic lesions identified as ICH were well-defined enough to reliably measure dimensions on cranial ultrasound.

#### Sample size estimation for future study to assess accuracy

Based on this exploratory study and the published literature in this area, (Additional file 2: Table S1) we determined that a total sample size of 310 patients (with 31 patients with ICH assuming the prevalence of ICH is 0.1 in this population) will achieve 81% power to detect a change in sensitivity from 0.7 to 0.9 using a two-sided binomial test, in other words ruling out a sensitivity of 70% (Null hypothesis) in favor of a sensitivity of 90% (or higher). In addition, there is 90% power to detect a change in specificity from 0.83 to 0.9 using a two-sided binomial test, in other words ruling out a specificity of 83% (Null Hypothesis) in favor of a specificity of 90% (or higher). The target significance level for these tests are 0.05 (two-sided). The actual significance level achieved by the sensitivity test is 0.0478 and achieved by the specificity test is 0.0378. (Additional file 3: Table S2).

## Discussion

Our study elucidates potential feasibility of using cranial POCUS as a screening test method to identify the presence of ICH. We uncovered several image acquisition principles that can inform a standardized protocol for B mode imaging for cranial indications across different presets with information on several anatomical structures that create identifiable hyperechoic signals. Our exploratory preliminary study supports the feasibility of ultrasound-based ICH diagnosis using POCUS and generated useful information to allow future sample size collection in designing such a study. Though not designed to investigate diagnostic accuracy, our sample showed a sensitivity of 100% (7/7, 95% CI 0.70–0.99), specificity of 50% (2/4, 95% CI 0.12–0.87) and accuracy of 81.82% (9/11, 95% CI 53–96%) when cranial ultrasound-based diagnosis was compared to CT/MRI-based diagnosis. We did not calculate predictive values due to small sample size and over-representation of ICH (7/11) in the sample compared to prevalence of this stroke subtype. Image acquisition principles and knowledge of ultrasound-based cranial topography gained from this study will be useful in informing future studies designed and powered to evaluate the sensitivity and specificity of this tool in ICH detection. Further characterization of the hyperechoic signals that cause false positive appearance of ICH and modulation of available presets to minimize false positive findings will be necessary to increase the specificity of this diagnostic tool. Based on this exploratory study, we anticipate a future pilot study on 310 patients designed to address the range of specificity and sensitivity of this tool.

Over 18 published studies (see Additional file 2: Table S1) performed in 590 patients including spontaneous ICH, traumatic ICH or hemorrhagic conversion of ischemic stroke have reported feasibility of ICH diagnosis using cranial B-mode ultrasound. However, all of these were performed more than a decade ago with high end ultrasound machines without detailed investigation into sensitivity, specificity or topography informing ICH diagnosis [2, 3, 5, 18, 19, 19–24]. We only found one relevant study assessing cranial ultrasound in pediatric head trauma that used POCUS equipment, though it did not elaborate on cranial topography [13]. Rapid accessibility to high-resolution CT scans and lack of sensitivity and specificity of ultrasound in detecting ischemic stroke (most stroke presentations) have prevented widespread adoption of this diagnostic modality despite evidence of early feasibility and reliability. Emerging technological advances in ultrasound have brought forth high resolution imaging in point of care devices typically used in critical care or emergency room settings, making this investigation relevant. Many clinical resuscitation algorithms in the pre-hospital, emergency and critical care settings have incorporated ultrasound; therefore, it is a natural progression for ultrasound to be tested for neurological emergencies, ICH being a critical one [25–27]. POCUS can be particularly useful in austere environments or in critically ill patients for serial monitoring, where bedside CT scan may not be available and critical equipment-like dialysis, extracorporeal circuits or advanced mechanical ventilation modes preclude safe transport. There is also increasing recognition that evaluation of time-based therapies for preventing early hematoma expansion in intracerebral hemorrhage have been challenging due to the lack of early field distinction of hemorrhagic and ischemic stroke. Published sensitivity and specificity for clinical scores to distinguish ischemic from hemorrhagic scores such as the Siriraj stroke score are 0.65, 0.88, and Guy's hospital stroke score are 0.54 and 0.89 for hemorrhagic stroke. [28, 29] The recent STOP_AUST trial showed favorable outcomes if patients received agents for reversal of anticoagulation in less than 3 h since onset highlighting the impact of early field diagnosis on interventions. [30] An imaging-based screening test such as cranial POCUS with or without combined clinical scores could provide an avenue to explore early field diagnosis of ICH and help in designing hyperacute trials to test early strategies, such as reversal of anticoagulation. Screening with ultrasound can also provide a cost-effective solution for screening nonspecific symptoms common in hemorrhagic stroke which may have slower onset or in clinical situations, where CT head is not amenable or available.

Our study has several limitations; a small sample size being the foremost. Our study recruitment was impacted by the pandemic-imposed restrictions which reduced availability of study staff and access to patients and their families for study procedures and consent. Our study was not designed to assess sensitivity, specificity, and accuracy analysis on cranial ultrasound in comparison with CT/MRI or selection of transcranial versus abdominal preset. However, by observing a sensitivity of 100% (7/7) with lower bounds of 95% confidence interval of 70% suggests that true sensitivity of this diagnostic approach warrants further exploration of cranial POCUS as a screening tool. With only 4 participants providing data toward specificity, we recognize need for larger studies for accurate determination of true specificity. Exploratory nature of study design and small sample size also prohibited meaningful analysis of impact of age, or comparison of CT Hounsfield units to sensitivity of ultrasound in detection of hemorrhage.

All scans were performed by one investigator with formal training in neurology and experience in neurovascular as well point of care ultrasound imaging. Current neurovascular training or critical care ultrasound training lacks any content relevant to cranial POCUS and is limited to transcranial doppler imaging and there is lack of any formal reference material of training for cranial POCUS B mode imaging. We used iterative learning with all studies performed by the single most qualified investigator with recognized national expertise (AS) in collaboration with neuroradiologists given formal training in neuroimaging. This model was used due to lack of published image library of cranial ultrasound pathology that informed learning of anatomical landmarks and artifacts on B mode ultrasound and further imaging. Final post hoc analysis was done with a neuroradiologist to inform on correlative anatomy described above. We found the imaging technique most useful in guiding future education on cranial B mode ultrasound imaging based on iterative scanning during this study.

Despite above limitations, our attempt at characterization of ultrasound-based diagnosis of ICH explored topography of anatomical structures on cranial B mode imaging that affect ultrasound-based ICH detection. We also performed all initial imaging with investigator blinded to intracerebral pathology diagnosis with post hoc comparison of ultrasound and CT scan to inform future imaging. One other published study with comparable sensitivity compared ultrasound imaging in patients with diagnosed intracerebral hemorrhage without blinding the ultrasonographer and used high end ultrasound machines. [31] Two other studies reporting 95% and 97.4 specificity were both done in a similar limited sample size on patients with known acute ischemic stroke with hemorrhagic transformation with unblinded sonographers.

This study has provided critical preliminary data to perform a larger clinical trial to assess the sensitivity, specificity, and diagnostic accuracy of cranial POCUS in detecting ICH (Cranial ultrasound for point of care detection of intracranial hemorrhage, CUPID_ICU) on individual presets that is supported by Wake Forest Neurosciences Clinical Trials Innovation Center and pre-hospital application of this modality in another study CUPID_EMS (ClinicalTrials.gov Identifier: NCT05492474) supported by Wake Forest Clinical and Translational Science Institute. Both these trials will rigorously investigate best ultrasound parameters and acquisition techniques to optimize ultrasound-based diagnosis of ICH using currently available handheld ultrasound machines. In addition, it will explore the learning curve of cranial ultrasound by training investigators naïve to ultrasound and/or neuroanatomy to explore the scalability of this investigation across different medical background and ultrasound expertise.

## Conclusions

Cranial POCUS has potential feasibility in use as a screening tool for diagnosis of ICH. Our exploratory analysis yields preliminary data on anatomical landmarks and artifacts seen in B mode cranial ultrasound imaging with guidance on optimal technique on the use of cranial ultrasound for ICH diagnosis. These findings will inform a future trial to explore the accuracy of cranial ultrasound compared to CT scan for possible field diagnosis of ICH.

## Supplementary Information

**Additional file 1: Figure S1.** Imaging Technique for Method of Insonation for Cranial Ultrasound.

**Additional file 2: Table S1.** Narrative summary of published studies investigating the accuracy of cranial ultrasound compared to CT in ICH diagnosis. AIS—acute ischemic stroke. NR-not reported or details not obtainable from data provided.

**Additional file 3: Table S2.** Sample size estimation based on One-Sample Sensitivity and Specificity Analysis.

## Data Availability

All data generated or analyzed during this study are included in this published article [and its supplementary information files].

## Abbreviations

CT: Computed tomography scan
MRI: Magnetic resonance image
IRB: Institutional review board
POCUS: Point of care ultrasound
ICH: Intracerebral hemorrhage
ICU: Intensive care unit
STROBE: Strengthening the reporting of observational studies in epidemiology
CI: Confidence intervals
