# Supplementary figures and images for: Exploratory study to assess feasibility of intracerebral hemorrhage detection by point of care cranial ultrasound
Source: Ultrasound J. 2022 Oct 17;14:40. doi: 10.1186/s13089-022-00289-z (PMC9576831; doi:10.1186/s13089-022-00289-z)

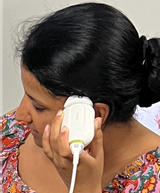

Supplement: Supplementary file 1 — Additional file 1: Figure S1. Imaging Technique for Method of Insonation for Cranial Ultrasound. [file 13089_2022_289_MOESM1_ESM.tif]
