# Supplementary material for: Exploratory study to assess feasibility of intracerebral hemorrhage detection by point of care cranial ultrasound
Source: Ultrasound J. 2022 Oct 17;14:40. doi: 10.1186/s13089-022-00289-z (PMC9576831; doi:10.1186/s13089-022-00289-z)
Supplement: Supplementary file 2 — Additional file 2: Table S1. Narrative summary of published studies investigating the accuracy of cranial ultrasound compared to CT in ICH diagnosis. AIS—acute ischemic stroke. NR-not reported or details not obtainable from data provided. [file 13089_2022_289_MOESM2_ESM.docx]

Supplemental material/Appendix: Table 4: Narrative summary of published studies investigating the accuracy of cranial ultrasound compared to CT in ICH diagnosis. AIS –acute ischemic stroke. NR-not reported or details not obtainable from data provided

| **Author** | **Study size (n)** | **Average age** | **Gender %male** | **% no temporal window** | **Detection Rate** | **Accuracy** | **Sensitivity** | **Specificity** | **False Positive** | **False Negative** | **US Machine** |
| --- | --- | --- | --- | --- | --- | --- | --- | --- | --- | --- | --- |
| Becker  1991 ^32^ | 29 ICH, 36 SAH, 38 brain tumors | 53.3 | 21 (58%) male, 36 total | 5 (14%) | 75% |  | NR | NR | NR | NR | Siemens Sonoline CF  2.25 MHz |
| Becker  1993 ^33^ | 48 (28 ICH, 20 AIS) | 55.3 | 31 (65%) male, 48 total | 6 (12.5%) | 24/28 (96%) | NR | NR | NR | NR | NR | Siemens Sonoline CF. 2.25 MHz transducer |
| Seidel  1993 ^34^ | 23 ICH (21 spont, 2 traumatic) | 66.4 | 14 (61%) male, 23 total | 3 (13%) | 19/23 (83%) | 18/23 (78%) | NR | NR | 1 | 1 | HP SONOS 1000  16cm depth |
| Seidel  1995 ^35^ | 84 AIS (15 ICH, ) | 62.9 | 48 (57%) male, 84 total | 17 (20%) | 14/15 (93%) | NR | 88% | 96% | 1 | 1 | HP SONOS 1000. 2.5 MHz  16 cm depth |
| Woydt  1996 ^36^ | 74 (35 spontaneous, 27 traumatic ICH, 12 excluded) | 55 | 46 (62%) male, 74 total | 12 (16%) | 39/42 ICH (92.9%), 18/19 EDH/SDH (95%) | NR | NR | NR | NR | 3 ICH, 1 in EDH/SDH | Siemens CF Sonoline |
| Lindner  1997 ^37^ | 9 ICH | 56 | 6 (67%) male, 9 total | 0 | 9 of 9 (100%) | NR | NR | NR | NR | NR | Siemens Sonoline CF2.25MHz |
| Maurer  1998 ^38^ | 151 - 60 ICH, 67 AIS, 24 other | 65.6 | 93 (62%) male, 151 total | 18 (12%) | 50 of 53 (94.3%) | 83% | 94% | 95% | 4 microangiopathy | 3 (2 parietal) | Seimens Sonoline Ellegra or Siemens AG, or Acuson 128 XP/4. 2.0 to 2.5 MHz transducer |
| Seidel  2005 ^39^ | 32 strokes (11 hemorrhagic conversions) | 57 | 20 (63%) male, 32 total | 1 of 33 (3%) | 10 of 11 (91%) | NR | 91% | 95% | 1 | 1 cortical parietal lobe | Philips SONOS 5500  2-4 MHz frequency, 16 cm depth |
| Kern  2008 ^40^ | 12 acute supratentorial ICH | 64.5 | 9 (75%) male, 12 total | 0 | 11/12 (92%) | NR | NR | NR | NR | NR | Philips HDI 5000  2-4MHz, 12cm depth, mechanical index 1.3 |
| Perez  2009 ^31^ | 59 ICH | NR | NR | 6 of 59 (10%) | 34 (100%) | NR | NR | NR | NR | NR | Hitachi EUB-2000  2MHz frequency |
| Seidel  2009 ^41^ | 55 AIS (20 hemorrhagic transformation of AIS) | 60.4 | 35 (64%) male, 55 total | 0 | 18 (90%) | NR | 90% | 97.40% | 1 | 2 small cortical HT | Philips SONOS 5500  2MHz and 7.5MHz frequency, 16 cm depth, |
| Matsumoto  2011^42^ | 48 ICH | 67.5 | 15 (75%) male, 20 total | 27 (56.3%) | 19/20 (95%) | NR | NR | NR | 0 | 1 | Philips HDI 5000  2.5MHz frequency |
| Kukulska 2012 ^43^ | 51 ICH | 67.5 | 26 (67%) male, 39 total | 12 (23.5%) | 34/39 (87%) | 97.10% | NR | NR | NR | 5 (12.8%) superficial cortical | Philips EnVisor  2.5MHz frequency |
| Oveson 2014 ^44^ | 44 (25 ICH) | 70.4 | 18 (72%) male, 25 total | 2 (4.5%) | NR |  | NR | NR | NR | NR | GE LOGIQ E9  1.7-3.1MHz |
| Camps-Renom 2017 ^45^ | 59 (35 ICH) | 72.2 | 23 (66%) male, 25 total | 5 (8%) | NR | NR | NR | NR | NR | NR | Philips CX50  2-5MHz, 14-16cm depth |
| Niesen 2018 ^46^ | 14 ICH | 66.4 | 10 (71%) male, 14 total | 0 | 14 of 14 (100%) | NR | NR | NR | NR | NR | GE Logique 7 expert  2-2.5Mhz, 16cm depth |
| Masaeli 2019 ^13^ | 538 head trauma  (37 ICH) | 5.6 | 295 (54%) male, 538 total | NR | 94.40% |  | <2yo: 85.7%  2-6yo: 80.0%  >6yo: 46.7% | <2yo: 98%  2-6yo: 98%  >6yo: 92.9% | <2yo: 3  2-6yo: 4  6-18yo: 11 | <2yo: 1  2-6yo: 3  6-18yo: 8 | SonoSite M-Turbo  2-5MHz |
| Niesen 2019 ^47^ | 40 supratentorial ICH | 66.3 | 26 (65%) male, 40 total | 0 | NR |  | NR | NR | NR | NR | GE Logique 7 expert  2-2.5Mhz, 16cm depth |

32. Becker G, Greiner K, Kaune B, et al. Diagnosis and monitoring of subarachnoid hemorrhage by transcranial color-coded real-time sonography. Neurosurgery 1991;28(6):814-20. (In eng). DOI: 10.1097/00006123-199106000-00005.

33. Becker G, Winkler J, Hofmann E, Bogdahn U. Differentiation between ischemic and hemorrhagic stroke by transcranial color-coded real-time sonography. J Neuroimaging 1993;3(1):41-7. (In eng). DOI: 10.1111/jon19933141.

34. Seidel G, Kaps M, Dorndorf W. Transcranial color-coded duplex sonography of intracerebral hematomas in adults. Stroke 1993;24(10):1519-27. (In eng). DOI: 10.1161/01.str.24.10.1519.

35. Seidel G, Kaps M, Gerriets T. Potential and limitations of transcranial color-coded sonography in stroke patients. Stroke 1995;26(11):2061-6. (In eng). DOI: 10.1161/01.str.26.11.2061.

36. Woydt M, Greiner K, Perez J, Becker G, Krone A, Roosen K. Transcranial duplex-sonography in intracranial hemorrhage. Evaluation of transcranial duplex-sonography in the diagnosis of spontaneous and traumatic intracranial hemorrhage. Zentralbl Neurochir 1996;57(3):129-35. (In eng).

37. Lindner A, Gahn G, Becker G. Transcranial duplex sonography of hyperacute intracerebral hemorrhages. J Neuroimaging 1997;7(3):199-202. (In eng). DOI: 10.1111/jon199773199.

38. Mäurer M, Shambal S, Berg D, et al. Differentiation between intracerebral hemorrhage and ischemic stroke by transcranial color-coded duplex-sonography. Stroke 1998;29(12):2563-7. (In eng). DOI: 10.1161/01.str.29.12.2563.

39. Seidel G, Cangür H, Albers T, Meyer-Wiethe K. Transcranial sonographic monitoring of hemorrhagic transformation in patients with acute middle cerebral artery infarction. J Neuroimaging 2005;15(4):326-30. (In eng). DOI: 10.1177/1051228405280174.

40. Kern R, Kablau M, Sallustio F, et al. Improved detection of intracerebral hemorrhage with transcranial ultrasound perfusion imaging. Cerebrovasc Dis 2008;26(3):277-83. (In eng). DOI: 10.1159/000147456.

31. Pérez ES, Delgado-Mederos R, Rubiera M, et al. Transcranial duplex sonography for monitoring hyperacute intracerebral hemorrhage. Stroke 2009;40(3):987-90. (In eng). DOI: 10.1161/strokeaha.108.524249.

41. Seidel G, Cangür H, Albers T, Burgemeister A, Meyer-Wiethe K. Sonographic evaluation of hemorrhagic transformation and arterial recanalization in acute hemispheric ischemic stroke. Stroke 2009;40(1):119-23. (In eng). DOI: 10.1161/strokeaha.108.516799.

42. Matsumoto N, Kimura K, Iguchi Y, Aoki J. Evaluation of cerebral hemorrhage volume using transcranial color-coded duplex sonography. J Neuroimaging 2011;21(4):355-8. (In eng). DOI: 10.1111/j.1552-6569.2010.00559.x.

43. Kukulska-Pawluczuk B, Książkiewicz B, Nowaczewska M. Imaging of spontaneous intracerebral hemorrhages by means of transcranial color-coded sonography. Eur J Radiol 2012;81(6):1253-8. (In eng). DOI: 10.1016/j.ejrad.2011.02.066.

44. Ovesen C, Christensen AF, Krieger DW, Rosenbaum S, Havsteen I, Christensen H. Time course of early postadmission hematoma expansion in spontaneous intracerebral hemorrhage. Stroke 2014;45(4):994-9. (In eng). DOI: 10.1161/strokeaha.113.003608.

45. Camps-Renom P, Méndez J, Granell E, et al. Transcranial Duplex Sonography Predicts Outcome following an Intracerebral Hemorrhage. AJNR American journal of neuroradiology 2017;38(8):1543-1549. (In eng). DOI: 10.3174/ajnr.A5248.

46. Niesen WD, Schläger A, Reinhard M, Fuhrer H. Transcranial Sonography to Differentiate Primary Intracerebral Hemorrhage from Cerebral Infarction with Hemorrhagic Transformation. J Neuroimaging 2018;28(4):370-373. (In eng). DOI: 10.1111/jon.12510.

13. Masaeli M, Chahardoli M, Azizi S, et al. Point of Care Ultrasound in Detection of Brain Hemorrhage and Skull Fracture Following Pediatric Head Trauma; a Diagnostic Accuracy Study. Arch Acad Emerg Med 2019;7(1):e53. (In eng).

47. Niesen WD, Schlaeger A, Bardutzky J, Fuhrer H. Correct Outcome Prognostication via Sonographic Volumetry in Supratentorial Intracerebral Hemorrhage. Front Neurol 2019;10:492. (In eng). DOI: 10.3389/fneur.2019.00492.
