# Supplementary material for: Exploratory study to assess feasibility of intracerebral hemorrhage detection by point of care cranial ultrasound
Source: Ultrasound J. 2022 Oct 17;14:40. doi: 10.1186/s13089-022-00289-z (PMC9576831; doi:10.1186/s13089-022-00289-z)

2/14/2022 12:19:41 PM 1

**One-Sample Sensitivity and Specificity Analysis**

**Numeric Results for testing H0: Se = Se0 vs. H1: Se ≠ Se0 and H0: Sp = Sp0 vs. H1: Sp ≠ Sp0**

**Test Statistic: Binomial Test**

**--- Sensitivity --- --- Specificity --- ------------- Alpha ------------ Preva-**

**----- Power ------ Sample Size H0 H1 H0 H1 Sens. Spec. lence**

**Sens. Spec. N1 and N Se0 Se1 Sp0 Sp1 Target Actual Actual P**

0.8068 0.9970 31 310 0.7000 0.9000 0.8000 0.9000 0.0500 0.0478 0.0429 0.1000

0.8068 0.8252 31 155 0.7000 0.9000 0.8000 0.9000 0.0500 0.0478 0.0320 0.2000

0.8068 0.9033 31 310 0.7000 0.9000 0.8300 0.9000 0.0500 0.0478 0.0378 0.1000

0.8068 0.5280 31 155 0.7000 0.9000 0.8300 0.9000 0.0500 0.0478 0.0306 0.2000

**References**

Obuchowski, N.A., Zhou, X.H. 2002. 'Prospective studies of diagnostic test accuracy when disease prevalence is

low,' Biostatistics, Volume 3, No. 4, pages 477-492.

Li, J., Fine, J. 2004. 'On sample size for sensitivity and specificity in prospective diagnostic accuracy

studies,' Statistics in Medicine, Volume 23, pages 2537-2550.

Machin, D., Campbell, M.J., Tan, S.B., Tan, S.H. 2008. Sample Size Tables for Clinical Studies, Third Edition.

Wiley-Blackwell, Chichester, United Kingdom.

Zhou, X.H., Obuchowski, N.A., McClish, D.K. 2002. Statistical Methods in Diagnostic Medicine.

Wiley-Interscience, New York.

**Report Definitions**

Sens. Power is the power of the sensitivity test. It is based on the N1 observations.

Spec. Power is the power of the specificity test. It is based on the N-N1 observations.

N is the total sample size of the study. It is equal to N1 + N2.

Se0 is the sensitivity under H0. The sensitivity is the proportion of diseased subjects that yield a positive

test result.

Se1 is the sensitivity under H1. The sensitivity is the proportion of diseased subjects that yield a positive

test result.

Sp0 is the specificity under H0. The specificity is the proportion of non-diseased subjects that yield a

negative test result.

Sp1 is the specificity under H1. The specificity is the proportion of non-diseased subjects that yield a

negative test result.

Target Alpha is the alpha (probability of rejecting H0 when H0 is true) that was desired.

Actual Sens. Alpha is the alpha that was actually achieved by the sensitivity test, calculated from the

binomial distribution.

Actual Spec. Alpha is the alpha that was actually achieved by the specificity test, calculated from the

binomial distribution.

P is proportion of the population that actually has the condition (disease) of interest, called the

prevalence.

**Summary Statements**

A total sample size of 310 (which includes 31 subjects with the disease) achieves 81% power to

detect a change in sensitivity from 0.7 to 0.9 using a two-sided binomial test and 100% power

to detect a change in specificity from 0.8 to 0.9 using a two-sided binomial test. The target

significance level is 0.05. The actual significance level achieved by the sensitivity test is

0.0478 and achieved by the specificity test is 0.0429. The prevalence of the disease is 0.1.

2/14/2022 12:19:44 PM 2

**One-Sample Sensitivity and Specificity Analysis**

**Sensitivity Chart Section**

2/14/2022 12:19:44 PM 3

**One-Sample Sensitivity and Specificity Analysis**


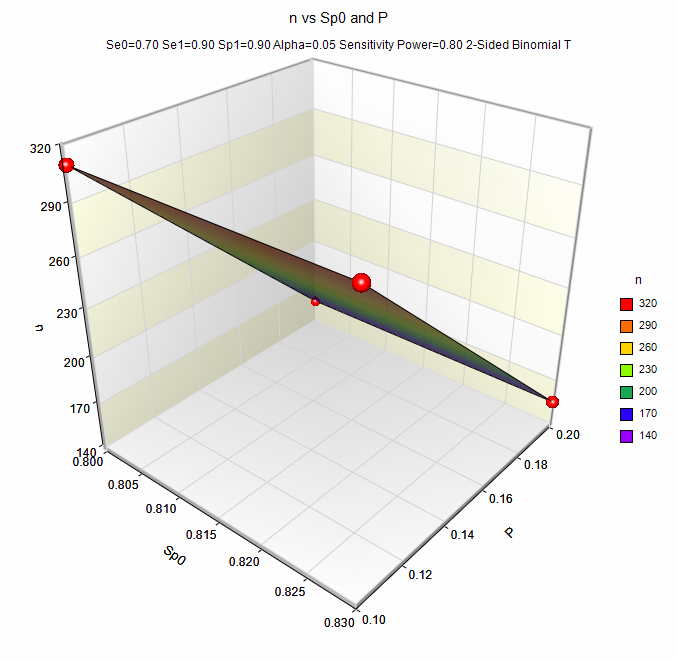


2/14/2022 12:19:44 PM 4

**One-Sample Sensitivity and Specificity Analysis**

**Specificity Chart Section**

2/14/2022 12:19:44 PM 5

**One-Sample Sensitivity and Specificity Analysis**


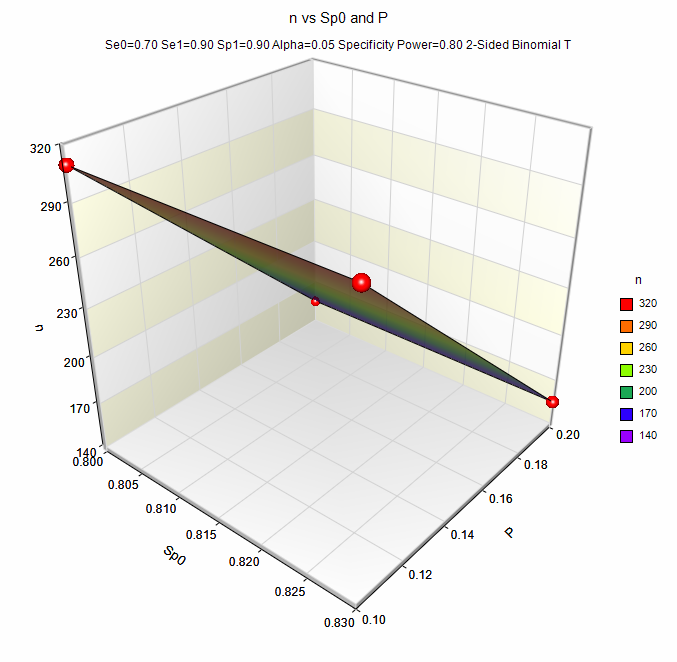

Supplement: Supplementary file 3 — Additional file 3: Table S2. Sample size estimation based on One-Sample Sensitivity and Specificity Analysis. [file 13089_2022_289_MOESM3_ESM.doc]
